# Physical activity promotion in persons with spinal cord injuries: Barriers and facilitators in low-resource communities

**DOI:** 10.4102/ajod.v11i0.988

**Published:** 2022-06-09

**Authors:** Candace Vermaak, Suzanne Ferreira, Elmarie Terblanche, Wayne Derman

**Affiliations:** 1 Division of Biokinetics, Department of Sport Science, Faculty of Medicine and Health Sciences, Stellenbosch University, Cape Town, South Africa; 2 Institute of Sport and Exercise Medicine, Department of Sport Science, Faculty of Health Sciences, Stellenbosch University, Cape Town, South Africa; 3 Division of Sport Science, Department of Sport Science, Faculty of Medicine and Health Sciences, Stellenbosch University, cape Town, South Africa; 4 International Olympic Committee Research Center, Cape Town, South Africa

**Keywords:** physical activity, spinal cord injury, barriers, facilitators, low resource communities

## Abstract

**Background:**

A spinal cord injury is a devastating and life-changing event that presents the affected individual with multiple challenges throughout life. Physical activity can help mitigate some of these challenges; however, in low-resource communities where opportunities for physical activity are scarce, these challenges are often exacerbated and multiple.

**Objective:**

This study aimed to identify the barriers and facilitators to physical activity, specifically in individuals with spinal cord injuries, in low-resourced communities.

**Methods:**

A total of 57 adults (> 20 years) with a spinal cord injury living in the Western Cape, South Africa completed the self-developed research questionnaire.

**Results:**

A total of 289 barriers and 290 facilitators were reported. The most frequently reported barriers were lack of transport (*n* = 35), impairment type (*n* = 32), lack of trained volunteers and appropriate programmes (*n* = 19 each) and lack of information received from therapists following discharge (*n* = 10). The most frequently reported facilitators were support from family (*n* = 38), the ‘enjoyment’ of physical activity and the fact that ‘it made me feel good’ (*n* = 37); safe and accessible facilities were reported by 25 participants and 12 participants reported that higher-quality programmes and better-trained staff would help them to be more physically active.

**Conclusion:**

Individuals with a spinal cord injury face many barriers in being physically active. Yet it is evident that people with spinal cord injuries in low-resourced communities are eager to participate and improve their health and physical function. However, this will only realise if practitioners reduce the barriers to access, provide relevant training to staff and volunteers, educate their patients about the importance of physical activity post discharge, and create tailored programmes in safe and accessible community facilities.

## Introduction

A spinal cord injury (SCI) is a devastating and life-changing neurological event with far-reaching impact on the lives of the patient, their family and caregivers (Livecchi 2011; Mothabeng 2011). Physical activity (PA) has the potential to mitigate the detrimental effects of a SCI, for example, by reducing musculoskeletal and neuropathic pain, increasing functional capacity and decreasing incidence of cardiovascular disease (Williams, Smith & Papathomas 2014). In addition, participation in PA alleviates the stigma experienced by persons with disabilities (PWDs) regarding competence (Martin 2013). Despite the benefits, people with a SCI (PWSCI) remain the most physically inactive segment of society (Williams et al. 2014). It is therefore imperative that the factors that play a role in the promotion of a physically active lifestyle in PWSCI be fully understood. People with a SCI require the supportive environment of a multidisciplinary team to guide them whilst they adapt to their disability. Whilst this supportive environment is usually present during inpatient rehabilitation and the early stages of postdischarge, there is a gap in the provision of services that can assist individuals with community reintegration, health and well-being (Vermaak 2016). This is particularly true in low-resource communities.

In South Africa, limited research has been conducted on PA in PWDs and specifically, in PWSCI. Spinal cord injuries are increasing in South Africa and are mainly caused by assault (60%) (Joseph≈et al. 2017). With the rise in incidence, there is also an improved rate of survival because of medical and technological advances; thus, there is an increased need for exercise rehabilitation services. However, South Africa has a limited number of rehabilitation facilities catering for PWSCI to facilitate the final phase of rehabilitation, namely community reintegration (Joseph et al. 2017). Health and fitness professionals have the potential to impact a large sector of the South African population that is underserved in terms of exercise rehabilitation or PA programmes.

An important first step is to understand the barriers and facilitators of PA. This is important not only in programme design and development but also in appropriate facility and community design that allows for the inclusion of PWSCI. The barriers to PA amongst PWD have been researched extensively; however, the majority of research has been conducted in developed countries (Kehn & Kroll 2009; Martin Ginis et al. 2008; Rimmer et al. 2004; Shakespeare & Kleine 2013; Vissers et al. 2008; Wright et al. 2019), yet 80% of the world’s population live in developing countries.

Furthermore, research on the facilitators of PA is limited as data are derived from developed countries (United States of America and Europe) and not necessarily translatable to the low-resourced environments in developing countries.

The aim of this study was thus to identify the barriers and facilitators of PA in PWSCI within a low-resourced South African context. To our knowledge, not much research has been conducted on the topic in Africa. In addition, the perspectives from the participants contribute to the existing body of knowledge available within this population. Moreover, the newly acquired knowledge can be applied in the development of strategies for future partnerships and programme development in an effort to ease the burden on an already pressurised healthcare system (Joseph et al. 2017) and contribute to a more inclusive society informed by the social model of disability.

## Method

### Study design

A descriptive research survey on PWSCI was conducted in the Western Cape province of South Africa. A questionnaire was designed based on an extensive review of the literature regarding barriers and facilitators to PA, as identified by PWSCI and PWD. The review included studies utilising focus groups (Rimmer et al. 2004), semi-structured interviews (Conchar et al. 2014), surveys (Scelza et al. 2005) and questionnaires (Jaarsma et al. 2014).

The research instrument and method of analysis were based on and developed from the theory of planned behaviour (TPB). Whilst many theories have been utilised within the context of PA participation and health behaviours to explain motivation and adherence (Gulley & Boggs 2014; McNeil, Kreuter & Subramanian 2006; Prochaska & Velicer 1997; Ryan & Patrick 2009), TPB was chosen for this study based on previous studies using similar categories.

The TBP involves two parts, namely consideration of the individual’s own attitudes towards the behaviour and consideration of relevant behavioural norms (Bozionelos & Bennett 1999). Personal attitudes are the measures of beliefs about the behaviour under consideration and the influence on those beliefs (Bozionelos & Bennett 1999). Factors that influence behaviour in this regard include obstacles or hindrances, experience, resources and opportunities.

Behavioural norms refer to the influence of others on a particular behaviour and the individual’s wish to comply with the expectations of others. This includes the opinions of family members and healthcare professionals, who both play an important role, as illustrated in the results and discussion section. These aspects have been taken into consideration when grouping the barriers and facilitators in the development of the research questionnaire and the data analysis.

The research questionnaire included sections on personal history, chronic and secondary conditions, injury, rehabilitation history and previous (prior to injury) and current PA levels, as well as reasons for being physically active (facilitators) and inactive (barriers). The majority of the questions were multiple choice, with some requiring ‘yes’ and ‘no’ answers. To solicit individual experiences and perspectives, space was provided on the questionnaire to capture any additional information regarding the barriers and facilitators identified by participants.

### Study population, research setting and data collection

The study population included PWSCIs residing in three low-to-middle-income communities within the Western Cape, namely Strand, Macassar and Mitchells Plain. The study participants were recruited from the Western Cape Rehabilitation Centre (a formal government rehabilitation setting) and Senecio (a nonprofit organisation) operating in low-to-moderate-income communities.

The questionnaire was piloted in four PWSCIs from different ethnic backgrounds. Minor changes were implemented based on the experience and feedback to the research team. Participants were recruited on a voluntary basis through identified agents, using convenience sampling. The exclusion criteria were persons with motor or sensory loss who had not been diagnosed with a SCI, participants who had not completed the informed consent form and participants who were younger than 18 years. A total of 57 participants completed the questionnaire.

### Data analysis

The data were captured on a spreadsheet and analysed using Excel 2007. Descriptive statistics regarding the sociodemographic and injury profile data were reported as frequencies and percentages. The data on the barriers and facilitators were also reported as frequencies and percentages of the total number of barriers or facilitators selected by the participants. The results were grouped according to the TPB into personal, environmental, social and programme or policy barriers and facilitators.

### Ethical considerations

The protocol was approved by the institutional Ethics Committee for Human Research (Humanoria HS1028/2014). All participants were required to provide informed consent. Information gathered from the questionnaires was kept confidential to protect participants’ identities. Hard copies of the completed questionnaires were scanned and saved in a password-protected file. Access to these questionnaires was only granted to the research team.

Ethical clearance to conduct this study was obtained from the Institutional Ethics Committee for Human research (Humanities), Stellenbosch University (No. REC-050411-032).

## Results and discussion

The sex distribution of the 57 participants was 49 men and 8 women (ratio 6:1). The average age of the participants was 38 (± 13) years, with the youngest participant being 20 and the oldest 84 years old. Most of the participants were between 20 and 30 years old (*n* = 15). The majority of the participants were paraplegic (35/61%) and most of the participants sustained their injuries through violence (44%). Gunshot or stab wounds (total 37%) represented most of the reported violent crimes. The causes for gunshot or stab wounds ranged from personal assault to robberies and gang violence. The remaining 7% were caused by blunt trauma. Motor vehicle accidents accounted for 19% of the injuries. Eleven percent of the injuries were caused by sporting accidents, including a diving incident, and 9% resulted from tuberculosis of the spine. Most of the participants were hospitalised within the Western Cape after their injury, with only 4% being hospitalised in the Eastern Cape. A total of 55 of the participants were hospitalised in government hospitals and only two PWSCI in a private hospital.

## Barriers to physical activity

A total of 289 barriers to PA were reported in this study and are shown in Table 1. The most common barriers were environmental (*n* = 101), followed by personal barriers (*n* = 95), programme or policy barriers (*n* = 73) and lastly social barriers (*n* = 20).

**TABLE 1:** Number and nature of reported barriers to physical activity.

Variables	*n*	%
**Environmental barriers**		
Lack of transport	35	35
Lack of facilities	32	32
Weather	17	17
Inaccessible facilities	9	9
Inconvenient location	6	5
Other	2	2
**Total**	**101**	**100**
**Personal barriers**		
Impairment type (Tetraplegic or paraplegic)	32	34(12/22)
Secondary conditions	16	17
Lack of skill	14	15
Lack of finances	13	14
Lack of knowledge	13	14
Lack of time	5	5
Other	2	2
**Total**	**95**	**100**
**Programme barriers**		
Lack of appropriate programmes	19	26
Lack of trained volunteers	19	26
Lack of adaptive equipment	14	19
Cost of programmes	10	14
Lack of staff capacity	8	11
Lack of guidance by staff	2	3
Negative attitudes by staff	1	1
Other	0	0
**Total**	**73**	**100**
**Social barriers**		
Lack of information received by therapists post discharge	10	50
Lack of family support	4	20
Lack of role models	3	15
Negative societal attitudes	2	10
Other	1	5
**Total**	**20**	**100**

*n*, total number of barriers.

### Environmental barriers

Environmental barriers constituted the largest group in this study (*n* = 101), of which lack of transport was identified as the most common barrier (35%). Another important barrier was the lack of suitable facilities for physical activities (32%), as well as inaccessibility of facilities (9%). The findings of this study are consistent with the international research from both adequately resourced (developed) and low-resourced (developing) environments. Lack of transport is consistently indicated as the primary barrier, followed by lack of accessibility or lack of facilities across all types of environments (Crawford & Stodolska 2008; Scelza 2005; Silver et al. 2012:105; The Life Group 2011).

South Africa, in general, lacks an extensive, efficient and safe public transport system. This affects PWD to a large extent and more specifically PWSCI, who are dependent on their wheelchairs for transport. This has an important ‘knock-on’ effect with respect to other variables becoming barriers to PA, such as the weather (17%), which is especially problematic during the rainy season and during summertime when day temperatures regularly exceed 30°C. Furthermore, adverse environmental conditions expose PWSCI to illnesses such as respiratory tract infections and skin conditions, for example, pressure sores over insensate areas, which are aggravated or infected by wet clothing. Inconvenient location of the facilities where programmes are offered was identified as a less important environmental barrier (7%); however, the lack of transport compounds this problem, as few individuals have the means to reach these locations independently. These challenges highlight the importance of adequate and accessible transport for PWSCI and further emphasise the additional barriers that PWSCI face, especially in low-resourced communities.

### Personal barriers

According to the study participants, the extent of their impairment was the primary personal barrier to participating in PA (34%). Most of the participants were paraplegics, which explains why this was the most reported barrier (22%). This finding is inconsistent with the literature, as, to our knowledge, no previous study reported impairment type as a barrier to PA (Tawashy et al. 2009). Perhaps our finding can be attributed to the participants’ perceptions and lack of knowledge about their abilities. Furthermore, it is possible that in a developing country, people perceive their own injury, rather than society and the environment, as a barrier, a view that emphasises the influence of the medical model of disability as compared with the social model.

Other reported barriers were lack of finances, lack of skill or fitness and lack of knowledge about PA, which are consistent with that reported in previous research (Crawford & Stodolska 2008; Rimmer et al. 2004; Shakespeare & Kleine 2013; Tasiemski et al. 2004). Interestingly, able-bodied adults in South Africa reported similar barriers to PA, namely lack of time, health issues and lack of knowledge (Jaarsma et al. 2014; Louw, Van Biljon & Mugandani 2012). Despite these common personal barriers, PWD face additional barriers, which should be taken into consideration when planning and implementing PA programmes.

Secondary medical conditions made up 17% of the reported personal barriers and included bladder infections (50%), pressure sores (31%) and pain (19%). These findings are consistent with the literature (Silver et al. 2012; Van Den Berg-Emons et al. 2008). Secondary conditions are often the cause of rehospitalisation, subsequent bed rest and further physical inactivity. It is therefore important that these barriers be eliminated to improve health and well-being of PWSCI (Silver et al. 2012).

In this study, lack of time was considered the least important personal barrier (5%). This finding is in contrast with observations in the uninjured population in South Africa (Jaarsma et al. 2014) and in PWSCI in countries with better resources. Conceivably, our finding may be a reflection of the high unemployment rate amongst the participants, which is a testament to the low labour market absorption of PWD in South Africa (Census 2011). O’Neil and Dyson-Hudson (2020) reported high variability in the employment rates of PWD. For example, in the United States of America, postinjury employment rates ranged from 21% to 67% (Lidal, Huynh & Biering-Sørensen 2007). Although none of the participants indicated employment as a barrier to PA, various barriers prevent PWSCI from working (O’Neil & Dyson-Hudson 2020).

### Programme or Policy barriers

The most common reported programme barrier in this study related to human interaction (41%). This included lack of trained volunteers (26%), lack of staff capacity (11%), lack of guidance by staff (3%) and negative attitudes by staff (1%). Other barriers mentioned were lack of appropriate programmes (26%), lack of adaptive equipment (19%) and cost of programmes (14%).

Most of the literature reports similar barriers, namely the cost of an exercise programme (Malone, Barfield & Brasher 2012; Scelza et al. 2005), lack of experience amongst fitness centre staff in working with PWDs (Scelza et al. 2005), lack of adaptive equipment (Crawford & Stodolska 2008; Rimmer & Henley 2013; The Life Group 2011), lack of training of coaches and community-based instructors (Johnson 2009), negative attitudes of healthcare professionals (Shakespeare & Kleine 2013) and the qualification of individuals who are responsible for supervision (Jaarsma et al. 2014). Although programme or policy barriers were the least reported barriers (13%), they remain important because PWSCI cannot participate safely in PA without suitable programmes, equipment and trained staff.

Many buildings and PA programmes in South Africa remain inaccessible to PWD and thus prohibit individuals from engaging in activities of daily living and being included in society. Unfortunately, the onus is on PWD rather than on society to seek alternatives or solutions. Many health disparities faced by PWD are not necessarily directly associated with the disability itself; rather, they reflect a lack of good health promotion practices, as well as environmental and social barriers (social model of disability) (Rimmer & Rowland 2008). Within the social model of disability, the following holds true: ‘[W]e were not disabled by our impairments but by the disabling barriers we faced in society’ (Oliver 2013:1024).

### Social barriers

The social barriers to PA identified in this study included lack of information (50%), lack of friend or family support (20%) and lack of role models (15%). This is consistent with previous research, which documented that participants do not receive adequate information regarding sporting opportunities post injury (Malone et al. 2012; Stephens, Neil & Smith 2012). Limited social support and family role functioning also contribute to the barriers (Magasi, Heinemann & Whiteneck 2008). In addition, many people do not believe that they may benefit from PA, because their doctors did not encourage them to be active (Scelza et al. 2005). Thus, health professionals should exploit their trusted positions as sources of information and discuss the importance of PA participation and its effect on overall well-being (Putnam et al. 2003) with their patients.

Negative societal attitudes were mentioned by 10% of the participants in this study and were also identified as one of the barriers in promoting health in PWD (Rimmer et al. 2008). The relatively low rate in this study can be ascribed to the fact that most of the participants were already physically active in areas that cater specifically for PWD. Therefore, they did not need to interact much with society around them when they were physically active and as a result did not experience negative societal attitudes.

## Facilitators to physical activity

Although PWSCI face many barriers to PA, many facilitators (*n* = 290) were also identified in the study and are summarised in Table 2.

**TABLE 2:** Reported facilitators to physical activity.

Variables	*n*	%
**Personal facilitators**		
Feel good, enjoy it	37	35
Desire to be active	35	33
Improve self-esteem	28	26
Other	6	6
**Total**	**106**	**100**
**Social facilitators**		
Family support	38	45
Positive encouragement from peers with a SCI	23	27
Role models	11	13
Positive societal attitudes	6	7
Adequate information	6	7
Other	0	0
**Total**	**84**	**100**
**Environmental facilitators**		
Accessible facilities	25	44
Facility in safe location	19	33
Transport	8	14
New facilities in rural areas	4	7
Other	1	2
**Total**	**57**	**100**
**Programme or policy facilitators**		
Better-quality programmes	12	28
Skilled staff	12	28
Trained volunteers	10	23
Enough staff	8	19
Other	1	2
**Total**	**43**	**100**

SCI, spinal cord injury.

*n*, total number of facilitators.

In this study, personal (37%) and social facilitators (29%) were the primary reasons for the participants’ PA participation. These facilitators concern the human aspect, involving the person directly or his or her social environment. The identified personal facilitators are associated with internal motivation, such as a desire to be active or to improve self-esteem. The social facilitators that emerged mainly concerned the people who supported them in being physically active. Although all facilitators are relevant, without a personal drive to be physically active or the necessary family support, PWSCI might not even leave their homes, much less attend a PA session.

### Personal facilitators

Personal facilitators were reported by participants (37%) as the main driver for being physically active. This group of factors is important, as it determines the person’s motivation to remain physically active. In this study, the most reported personal facilitator was firstly that people enjoyed exercise and that it made them feel good (35%). Secondly, they had a desire to be physically active (33%). Some of the participants also declared that they were physically active to improve their self-confidence (26%). These facilitators correspond with those reported in previous research (Bailey et al. 2013; Stephens et al. 2012). Similar facilitators were also identified in the able-bodied population, where the participants agreed that feeling good motivated them to continue to or want to be physically active (Louw et al. 2012).

Similar to an able-bodied population (Louw et al. 2012), the participants in this study expressed the desire to improve general health and strength as a facilitator. In SCI participants, researchers found that having fun and improving physical fitness and strength were great motivators in well-resourced environments (Kehn & Kroll 2009; Wu & Williams 2001), which is consistent with the results of this study. Whilst the prevention of secondary health conditions is an important facilitator in well-resourced environments, especially in the SCI community (Kehn & Kroll 2009), it was not mentioned by participants in this study as one of the facilitators. Yet, the majority of the participants acknowledged in the questionnaire that PA helps to prevent secondary conditions.

Previous research also identified that participants felt they had to prove themselves (Stephens et al. 2012). This is frequently mentioned in the literature as a reason why PWD participate in competitive sports (Huang & Brittain 2006; Page, O’Connor & Peterson 2001). However, this did not emerge as a facilitator in this study. It is evident that people engage in PA for various reasons and that for some, early engagement is necessary for a positive self-identity and for others, it only becomes important at a later stage (Levins, Redenbach & Dyck 2014). Regardless of the timing, PWSCI want to be physically active and should be afforded opportunities to do so.

### Social facilitators

Most of the participants in this study (45%) highlighted family and friend support as a facilitator. This was also a key facilitator in previous literature from well-resourced environments (Keegan et al. 2012; Stephens et al. 2012), as it facilitates personal commitment (Keegan et al. 2012). In addition, support from peers with disabilities was also identified as a facilitator, which encourages commitment to PA (Jaarsma et al. 2014). This also includes having role models amongst physically active people (Page et al. 2001; Stephens et al. 2012). In this study, these factors were reported by 27% (peers) and 13% (role models) of the participants.

As the first point of contact after acquiring an injury, rehabilitation and medical staff are considered as a crucial facilitator, as PWSCI highly depend on information they receive from the professionals. Surprisingly, this facilitator accounted for merely 7% of the social facilitators identified in the study, indicating that PWSCI are not receiving adequate information regarding their health and PA opportunities before they are discharged from hospital. Another infrequently reported facilitator of PA behaviour was positive societal attitudes (7%). This low percentage possibly indicates that there may still be a stigma associated with PWD in South Africa, which is consistent with the findings from well-resourced environments (Rimmer & Rowland 2008).

### Environmental facilitators

Environmental facilitators are important, especially since they are associated with accessibility. For the majority of PWD, including PWSCI, accessibility is a major issue because of their decreased or limited mobility. Unsurprisingly, therefore, accessible facilities were the most frequently reported facilitator in this study (44%). Even though people with health disparities associated with a disability, such as a SCI, have a tendency to live hypoactive lifestyles, they have the right to be physically active (Riley et al. 2008), and for this they require accessible facilities. Many secondary health conditions can be prevented or minimised through PA, whilst lack of access to facilities exacerbates the disability through its effect on a person’s health status. The latter predisposes PWD to remain homebound, which may exacerbate sedentary behaviour and caloric intake (Rimmer & Rowland 2008). These behaviours can ultimately lead to obesity and additional or other secondary conditions, which further intensifies the barriers they face.

Safe locations (33%) also emerged as an important environmental facilitator. This is understandable because PWD, including PWSCI, are vulnerable and easy targets for crime, especially in South Africa. According to the National Crime Victims’ Rights Week (NCVRW) Resource Guide (2015) and the World Health Organisation (WHO) Department of Violence and Injury Prevention and Disability (2015), PWD are far more often victims of crimes than the rest of the population. Hence, safe locations of facilities are essential when catering for PWSCI. Whilst transport was another facilitator reported to increase PA participation, it was less frequently indicated in this study. Most PWSCI in South Africa do not drive themselves, have a vehicle or have access to public transport in comparison to well-resourced environments.

### Programme or Policy facilitators

Most of the facilitators identified in the programme or policy section were related to people being available and competent to assist, such as skilled staff (28%), enough staff (19%) and sufficient number of trained volunteers (23%). Having sufficient and skilled staff is important as some PWSCI need assistance with wheelchair transfers, stretching and guidance by staff in order to execute exercises correctly.

Lastly, the availability of more and better-quality programmes that are tailored to individual needs (28%) was also highlighted. Although only a few studies reported on programme or policy facilitators, it was found that individually tailored programmes, a facility that supports people with similar conditions and disabilities and an exercise programme that considers individual motivators (The Life Group 2011) are considered facilitators in well-resourced environments. Surprisingly, very few researchers reported on this vital facilitator, yet without trained staff and appropriate programmes, PWSCI will not be able to partake in safe and healthy behaviours, which includes PA. Nevertheless, this might be a differentiating factor between well-resourced and under-resourced environments because under-resourced environments have fewer opportunities for PWD in comparison to developed countries, where trained staff and appropriate programmes are almost guaranteed.

## Strengths and limitations

The results of this study must be interpreted in the context of its limitations. The limitations include the small number of participants that completed the questionnaire. Moreover, participants were limited to individuals living within the Western Cape, one of nine provinces in South Africa. Additional or other barriers may be experienced by PWSCI living in other parts of the country, requiring alternative interventions and different facilitators. Another aspect to consider is sampling bias. The majority of the participants were either involved or were previously involved in some form of PA, thus their perspectives towards PA participation may be biased and not representative of the entire population of PWD in the Western Cape. It is, however, of great concern that the majority of the PWD, including PWSCI, do not have any access to facilities and programmes that provide PA opportunities and therefore the barriers (and facilitators) reported could be underestimated in terms of both importance and impact.

## Implications

Physical activity for PWSCI is a multifaceted issue and further research needs to be conducted to understand the predictors of PA participation (Tawashy et al. 2009) and continued participation. This study needs to be replicated in a larger and more diverse sample. This includes PWSCI from all nine provinces within South Africa. Subsequent to the proposed expanded study, the design of a national intervention should be tailored to the specific needs of PWSCI and implemented within different communities in order to determine what is still required for community-based health and wellness. Therefore, future research is also encouraged to better understand the change in barriers that takes place once an intervention has been implemented and to determine whether a community-based PA programme is sustainable over a long period of time (> 12 months). Furthermore, future research might be supplemented by qualitative studies that focus on the lived experiences of PWSCI regarding the barriers and facilitators in order to add depth to this study. A better understanding is required for the development and implementation of PA programmes promoting health and wellness within PWSCI by reducing the identified barriers and enhancing the facilitators. Lastly, the study can be replicated in groups of people who have acquired other disabilities.

## Conclusion

It is evident that PWSCI face various obstacles in being physically active. However, in low-resource environments, these barriers are often multiple and include a combination of personal, environmental, social and programme barriers. When physical inactivity is not addressed within PWSCI, health and wellness are not achieved, quality of life is affected and affected individuals are predisposed to many other comorbidities and secondary health conditions. The latter place a strain not only on the individual and family but also on national economies, especially in low-income to middle-income countries. Physical activity is a modifiable risk factor with many health benefits which all are entitled to experience and ought to have access to.

## References

[CIT0001] Bailey, R., Hillman, C., Arent, S. & Petitpas, A., 2013, ‘Physical activity: An underestimated investment in human capital?’, *Journal of Physical Activity and Health* 10(3), 289–308. 10.1123/jpah.10.3.289 23620387

[CIT0002] Bozionelos, G. & Bennett, P., 1999, ‘The theory of planned behaviour as predictor of exercise: The moderating influence of beliefs and personality variables’, *Journal of Health Psychology* 4(4), 517–529. 10.1177/135910539900400406 22021644

[CIT0003] Conchar, L., Bantjes, J., Swartz, L. & Derman, W., 2014, ‘Barriers and facilitators to participation in physical activity: The experiences of a group of South African adolescents with cerebral palsy’, *Journal of health psychology* 21(2), 152–163. https://doi:10.1177/1359105314523305. 24607923 10.1177/1359105314523305

[CIT0004] Crawford, J.L. & Stodolska, M., 2008, ‘Constraints experienced by elite athletes with disabilities in Kenya, with implications for the development of a new hierarchical model of constraints at the societal level’, *Journal of Leisure Research* 40(I), 128–155. 10.1080/00222216.2008.11950136

[CIT0005] Department: Statistics South Africa, 2015, *Census 2011: Profile of persons with disabilities in South Africa*, viewed 29 June 2015, from http://beta2.statssa.gov.za/publications/Report-03-01-59/Report-03-01-592011.pdf.

[CIT0006] Gulley, T. & Boggs, D., 2014, ‘Time perspective and the theory of planned behavior: Moderate predictors of physical activity among central Appalachian adolescents’, *Journal of Pediatric Health Care* 28(5), 41–47. 10.1016/j.pedhc.2014.02.009 24793985

[CIT0007] Huang, C.J. & Brittain, I., 2006, ‘Negotiating identities through disability sport’, *Sociology of Sport Journal* 23(4), 352–375. 10.1123/ssj.23.4.352

[CIT0008] Jaarsma, E.A, Dijkstra, P.U., Geertzen, J.H.B. & Dekker, R., 2014, ‘Barriers to and facilitators of sports participation for people with physical disabilities: A systematic review’, *Scandinavian Journal of Medicine and Science in Sports* 24(5), 1–11. 10.1111/sms.12218 24730752

[CIT0009] Jacobs, P.L. & Nash, M.S., 2004, ‘Exercise recommendations for individuals with spinal cord injury’, *Sports Medicine* 34(11), 727–751. 10.2165/00007256-200434110-00003 15456347

[CIT0010] Johnson, C.C., 2009, ‘The benefits of physical activity for youth with developmental disabilities: A systematic review’, *American Journal of Health Promotion* 23(3), 157–167. 19149420 10.4278/ajhp.070930103

[CIT0011] Joseph, C., Scriba, E., Wilson, V., Mothabeng, J. & Theron, F., 2017, ‘People with spinal cord injury in Republic of South Africa’, *American Journal of Physical Medicine & Rehabilitation* 96(2), 109–111. 10.1097/PHM.0000000000000594 28059894

[CIT0012] Keegan, J.P., Chan, F., Ditchman, N & Chiu, C.Y., 2012, ‘Predictive ability of Pender’s health promotion model for physical activity and exercise in people with spinal cord injuries’, *Rehabilitation Counselling Bulletin* 56(1), 34–47. 10.1177/0034355212440732

[CIT0013] Kehn, M. & Kroll, T., 2009, ‘Staying physically active after spinal cord injury: A qualitative exploration of barriers and facilitators to exercise participation’, *BMC Public Health* 1(9), 168–178. 10.1186/1471-2458-9-168 PMC2694784 19486521

[CIT0014] Levins, S.M., Redenbach, D.M. & Dyck, I., 2004, ‘Individual and societal influences on participation in physical activity following spinal cord injury: A qualitative study’, *Physical Therapy* 84(6), 496–509. 10.1093/ptj/84.6.496 15161416

[CIT0015] Lidal, I.B., Huynh, T.K. & Biering-Sørensen, F., 2007, ‘Return to work following spinal cord injury: A review’, *Disability and Rehabilitation* 15(29), 1341–1375. 10.1080/09638280701320839 17729082

[CIT0016] Livecchi, M.A., 2011, ‘Spinal cord injury’, *Continuum* 17(3), 568–583. 10.1212/01.CON.0000399073.00062.9e 22810868

[CIT0017] Louw, A., Van Biljon, A. & Mugandani, S., 2012, ‘Exercise motivation and barriers among men and women of different age groups: Sport psychology’, *African Journal for Physical Health Education Recreation and Dance*, 18(4), 759–768.

[CIT0018] Magasi, S.R., Heinemann, A.W. & Whiteneck, G.G., 2008, ‘Participation following traumatic spinal cord injury: An evidence-based review for research’, *The Journal of Spinal Cord Medicine* 31(2), 145–146. 10.1080/10790268.2008.11760705 18581661 PMC2565477

[CIT0019] Malone, L.A., Barfield, J.P. & Brasher, J.D., 2012, ‘Perceived benefits and barriers to exercise among persons with physical disabilities or chronic health conditions within action or maintenance stages of exercise’, *Disability and Health Journal* 5(4), 254–260. 10.1016/j.dhjo.2012.05.004 23021736

[CIT0020] Martin-Ginis, K.A., Latimer, A.E., Buchholz, A.C., Bray, S.R., Craven, B.C., Hayes, K.C., et al., 2008, ‘Establishing evidence-based physical activity guidelines: Methods for the study of health and activity in people with spinal cord injury (SHAPE SCI)’, *Spinal Cord* 46(3), 216–221. 10.1038/sj.sc.3102103 17646838

[CIT0021] Martin, J., 2013, ‘Benefits and barriers to physical activity for individuals with disabilities: A social-relational model of disability perspective’, *Disability and Rehabilitation* 35(24), 2030–2037. 10.3109/09638288.2013.802377 23781907

[CIT0022] McNeil, L.H., Kreuter, M.W. & Subramanian, S.V., 2006, ‘Social environment and physical activity: A review of concepts and evidence’, *Social Sciences and Medicine* 63(4), 1011–1022. 10.1016/j.socscimed.2006.03.012 16650513

[CIT0023] Mothabeng, D.J., 2011, ‘Community participation for people living with spinal cord injury in the Tshwane metropolitan area’, PhD thesis, Dept. of Physiotherapy, University of Pretoria.

[CIT0024] O’Neil, J. & Dyson-Hudson, T.A., 2020, ‘Employment after spinal cord injury’, *Current Physical Medicine and Rehabilitation Reports* 8, 141–148. 10.1007/s40141-020-00266-4

[CIT0025] Oliver, M., 2013, ‘The social model of disability: Thirty years on’, *Disability and Society* 28(7), 1024–1026. 10.1080/09687599.2013.818773

[CIT0026] Page, S.J., O’Connor, E. & Peterson, K., 2001, ‘Leaving the disability ghetto: A qualitative study of factors underlying achievement motivation among athletes with disabilities’, *Journal of Sport and Social Issues* 25(1), 40–55. 10.1177/0193723501251004

[CIT0027] Papathomas, A., Williams, T.L. & Smith, B., 2015, ‘Understanding physical activity participation in spinal cord injured populations: Three narrative types for consideration’, *International Journal of Qualitative Studies on Health and Wellbeing* 10(1), 1–12. 10.3402/qhw.v10.27295 PMC4539383 26282868

[CIT0028] Parschau, L., Richert, J., Koring, M., Ernsting, A., Lippke, S. & Schwarzer, R., 2012, ‘Changes in social-cognitive variables are associated with stage transitions in physical activity’, *Health Education Research* 27(1), 129–140. 10.1093/her/cyr085 21890843

[CIT0029] Prochaska, J.O. & Velicer, W.F., 1997, ‘The transtheoretical model of health and behaviour’, *American Journal of Health Promotion* 12(1), 8–48. 10.4278/0890-1171-12.1.38 10170434

[CIT0030] Putnam, M., Greenen, S., Powers, L., Saxton, M., Finey, S. & Dautel, P., 2003, ‘Health and wellness: People with disabilities discuss barriers and facilitators to well-being’, *Journal of Rehabilitation* 69(1), 37–45.

[CIT0031] Riley, B.B., Rimmer, J.H., Wang, E. & Schiller, W.J., 2008, ‘A conceptual framework for improving the accessibility of fitness and recreation facilities for people with disabilities’, *Journal of Physical Activity and Health* 5(1), 158–168. 10.1123/jpah.5.1.158 18209261

[CIT0032] Rimmer, J.H. & Henley, K.Y., 2013, ‘Building the crossroad between inpatient/outpatient rehabilitation and lifelong community-based fitness for people with neurologic disability’, *Journal of Neurologic Physical Therapy* 37(2), 72–77. 10.1097/NPT.0b013e318291bbf6 23681276

[CIT0033] Rimmer, J.H. & Rowland, J., 2008, ‘Health promotion for people with disabilities: Implications for empowering the person and promoting disability-friendly environments’, *American Journal of Lifestyle Medicine* 2(5), 5409–5420. 10.1177/1559827608317397

[CIT0034] Rimmer, J.H., Ainsworth, B., Young, D. & La Monte, M., 2008, ‘Promoting inclusive physical activity communities for people with disabilities’, *President’s Council on Physical Fitness and Sports Research Digest* 9(2), 1–8.

[CIT0035] Rimmer, J.H., Riley, B., Wang, E., Rauworth, A. & Jurkowski, J., 2004, ‘Physical activity participation among persons with disabilities: Barriers and facilitators’, *American Journal of Preventive Medicine* 26(5), 419–425. 10.1016/j.amepre.2004.02.002 15165658

[CIT0036] Ryan, R.M. & Patrick, H., 2009, ‘Self-determination theory and physical’, *Hellenic Journal of Psychology* 6(2), 107–124.

[CIT0037] Scelza, W.M., Kalpakjian, C.Z., Zemper, E.D. & Tate, D.G., 2005, ‘Perceived barriers to exercise in people with spinal cord injury’, *American Journal of Physical Medicine and Rehabilitation* 84(8), 576–583. 10.1097/01.phm.0000171172.96290.67 16034226

[CIT0038] Shakespeare, T. & Kleine, I., 2013, ‘Educating health professionals about disability: A review of interventions’, *Health and Social Care Education* 2(2), 20–37. 10.11120/hsce.2013.00026

[CIT0039] Silver, J., Ljungberg, I., Libin, A. & Groah, S., 2012, ‘Barriers for individuals with spinal cord injury returning to the community: A preliminary classification’, *Disability and Health Journal* 5(3), 190–196. 10.1016/j.dhjo.2012.03.005 22726860

[CIT0040] Stephens, C., Neil, R. & Smith, P., 2012, ‘The perceived benefits and barriers of sport in spinal cord injured individuals: A qualitative study’, *Disability and Rehabilitation* 34(24), 2061–2070. 10.3109/09638288.2012.669020 22494335

[CIT0041] Tasiemski, T., Kennedy, P., Gardner, B.P. & Taylor, N., 2004, ‘The association of sports and physical recreation with life satisfaction in a community sample of people with spinal cord injuries’, *Neuro Rehabilitation* 20(4), 53–265. 10.3233/NRE-2005-20403 16403994

[CIT0042] Tawashy, A.E., Eng, J.J., Lin, K.H., Tang, P.F. & Hung, C., 2009, ‘Physical activity is related to lower levels of pain, fatigue and depression in individuals with spinal-cord injury: A correlational study’, *Spinal Cord* 47(4), 301–306. 10.1038/sc.2008.120 18936771 PMC3095632

[CIT0043] The Life Group, 2011, ‘Supporting community-based exercise in long-term neurological conditions: Experience from the Long-term Individual Fitness Enablement (LIFE) project’, *Clinical Rehabilitation* 25(7), 579–587. 10.1177/0269215510392075 21697208

[CIT0044] Van den Berg-Emons, R.J., Bussmann, J.B., Haisma, J.A., Sluis, T.A., Van der Woude, L.H., Bergen, M.P., et al., 2008, ‘A prospective study on physical activity levels after spinal cord injury during inpatient rehabilitation and the year after discharge’, *Archives of Physical Medicine and Rehabilitation* 89(11), 2094–2101. 10.1016/j.apmr.2008.04.024 18996237

[CIT0045] Vermaak, C., 2016, Bridging the gap from inpatient rehabilitation to sustainable health and wellness in spinal cord injured individuals [unpublished PhD thesis]. Stellenbosch University.

[CIT0046] Vissers, M., Van den Berg-Emons, R., Sluis, T., Bergen, M., Stam, H. & Bussmann, H., 2008, ‘Barriers to and facilitators of everyday physical activity in persons with a spinal cord injury after discharge from the rehabilitation centre’, *Journal of Rehabilitation Medicine* 40(6), 461–467. 10.2340/16501977-0191 18509562

[CIT0047] Williams, T.L., Smith, B. & Papathomas, A., 2014, ‘The barriers, benefits and facilitators of leisure time physical activity among people with spinal cord injury: A meta-synthesis of qualitative findings’, *Health Psychology Review* 8(4), 404–425. 10.1080/17437199.2014.898406 25211208

[CIT0048] Williams, T.L., Smith, B. & Papathomas, A., 2018, ‘Physical activity promotion for people with spinal cord injury: Physiotherapists’ beliefs and actions’, *Disability and Rehabilitation* 40(1), 52–61. 10.1080/09638288.2016.1242176 27917686

[CIT0049] World Health Organisation (WHO), 2015, *Physical activity*, viewed 13 September 2015, from http://www.who.int/dietphysicalactivity/pa/en/.

[CIT0050] World Health Organisation (WHO), 2020, *Physical inactivity a leading cause of disease and disability, warns WHO*, viewed 17 November 2020, from https://www.who.int/news/item/04-04-2020-physical-inactivity-a-leading-cause-of-disease-and-disability-warns-who. 12365404

[CIT0051] Wright, A., Roberts, R., Bowman, G. & Crettenden, A., 2019, ‘Barriers and facilitators to physical activity participation for children with physical disability: Comparing and contrasting the views of children, young people, and their clinicians’, *Disability and Rehabilitation* 41(13), 1499–1507. 10.1080/09638288.2018.1432702 29382235

[CIT0052] Wu, S.K. & Williams, T., 2001, ‘Factors influencing sport participation among athletes with spinal cord injury’, *Medicine and Science in Sports and Exercise* 33(2), 177–181. 10.1097/00005768-200102000-00001 11224802

